# Assessing clinical reasoning in undergraduate medical students during history taking with an empirically derived scale for clinical reasoning indicators

**DOI:** 10.1186/s12909-020-02260-9

**Published:** 2020-10-19

**Authors:** Sophie Fürstenberg, Tillmann Helm, Sarah Prediger, Martina Kadmon, Pascal O. Berberat, Sigrid Harendza

**Affiliations:** 1 Department of Internal Medicine, University Medical Center Hamburg-Eppendorf, III Medizinische Klinik, Martinistr. 52, D-20246 Hamburg, Germany; 2 Faculty of Medicine, University of Augsburg, Deanery, Augsburg, Germany; 3 TUM Medical Education Center, School of Medicine, Technical University of Munich, Munich, Germany

**Keywords:** Assessment, Clinical reasoning, Competence, History taking, Medical education

## Abstract

**Background:**

The clinical reasoning process, which requires biomedical knowledge, knowledge about problem-solving strategies, and knowledge about reasons for diagnostic procedures, is a key element of physicians’ daily practice but difficult to assess. The aim of this study was to empirically develop a Clinical Reasoning Indicators-History Taking-Scale (CRI-HT-S) and to assess the clinical reasoning ability of advanced medical students during a simulation involving history taking.

**Methods:**

The Clinical Reasoning Indictors-History Taking-Scale (CRI-HT-S) including a 5-point Likert scale for assessment was designed from clinical reasoning indicators identified in a qualitative study in 2017. To assess indicators of clinical reasoning ability, 65 advanced medical students (semester 10, *n* = 25 versus final year, *n* = 40) from three medical schools participated in a 360-degree competence assessment in the role of beginning residents during a simulated first workday in hospital. This assessment included a consultation hour with five simulated patients which was videotaped. Videos of 325 patient consultations were assessed using the CRI-HT-S. A factor analysis was conducted and the students’ results were compared according to their advancement in undergraduate medical training.

**Results:**

The clinical reasoning indicators of the CRI-HT-S loaded on three factors relevant for clinical reasoning: 1) focusing questions, 2) creating context, and 3) securing information. Students reached significantly different scores (*p* < .001) for the three factors (factor 1: 4.07 ± .47, factor 2: 3.72 ± .43, factor 3: 2.79 ± .83). Students in semester 10 reached significantly lower scores for factor 3 than students in their final year (*p* < .05).

**Conclusions:**

The newly developed CRI-HT-S worked well for quantitative assessment of clinical reasoning indicators during history taking. Its three-factored structure helped to explore different aspects of clinical reasoning. Whether the CRI-HT-S has the potential to be used as a scale in objective structured clinical examinations (OCSEs) or in workplace-based assessments of clinical reasoning has to be investigated in further studies with larger student cohorts.

## Background

Clinical reasoning is a core element of medical practice. In medical experts, the thinking during a patient contact happens with the most likely diagnostic hypothesis being formed within the first minutes [1]. This hypothesis is confirmed, refined or ruled out as a result of further information through specific target-oriented questions [1]. While experts begin the clinical reasoning process by intuitively generating a list of hypotheses using their experiential knowledge [2], novices seem to consciously match patients’ symptoms to the concepts they have memorized [3]. Additionally, contextual factors of patients’ histories appear to have an impact on medical experts’ clinical reasoning performance [4, 5]. Because some clinical reasoning skills are either subconscious or not fully articulated by clinicians [6] they are difficult to be taught [7] and often acquired in an informal rather than an explicit way, e.g. during clerkships or bedside teaching. After a patient encounter, the teacher often asks medical students only for a possible diagnosis and diagnostic tests leading to this diagnosis without considering the reasoning process itself [8].

Assessment of clinical reasoning is also challenging because it must be inferred from behaviour [9]. Different assessment strategies have been suggested [9, 10] and their combination is recommended to assess diagnostic accuracy with respect to different content and contexts [9]. We may have some clues to how novices articulate and enact clinical reasoning during history-taking based on qualitative observations by Haring et al. [11]. In their study, medical expert watched recorded medical students’ history taking in patient encounters and discussed their discoveries whenever they felt clinical reasoning occurred [11]. These qualitatively identified clinical reasoning indicators consist of general and specific observable phenomena which include context factors and frames of reference incorporated by the assessors [12]. Furthermore, the experts also considered themselves as a reference for clinical reasoning, being able to observe how well students can engage in hypothesis directed data gathering during history taking [11]. This is in line with the social perception theory assuming that assessors use idiosyncratic pre-existing schemes based on expectations in the evaluation of students in a specific situation [13]. It is not fully understood how such internal frames or standards to assess clinical reasoning are developed [14].

Since Haring et al. identified indicators for clinical reasoning [11], the aim of our study was to develop an instrument that incorporates these expert-based indicators to assess medical students’ clinical reasoning during simulated patient consultations in an attempt to standardize assessors’ observations of clinical reasoning. The quality of this new instrument to assess medical students’ clinical reasoning by observing their history taking was defined and the clinical reasoning ability of medical students of different advancement in their undergraduate studies was assessed.

## Methods

To assess indicators of clinical reasoning ability advanced medical students participated in a 360-degree competence assessment in the role of beginning residents during a simulated first workday of residency [15]. This assessment, representing a high-fidelity simulation of a clinical environment, was based on selected competences relevant for beginning residents [16]. The assessment consisted of three phases: 1) a consultation hour with five simulated patients, 2) a management phase (2.5 h) to organize these patients’ next diagnostic steps and to interact with other health care personnel, and 3) a handover of the patients to a resident (30 min). The consultation hour with five simulated patients covering five different contexts, namely a 42-year-old female with palpitations (patient 1), a 53-year-old male with fatigue and hemoptysis (patient 2), a 58-year-old female with abdominal pain (patient 3), a 54-year-old male with flank pain (patient 4), and a 36-year-old female with rheumatoid arthritis and fever (patient 5), as well as the handover were video-recorded. The patient cases were based on real patients and designed with elements of complexity that were intended to induce analytic thinking processes [17].

**Fig. 1:**
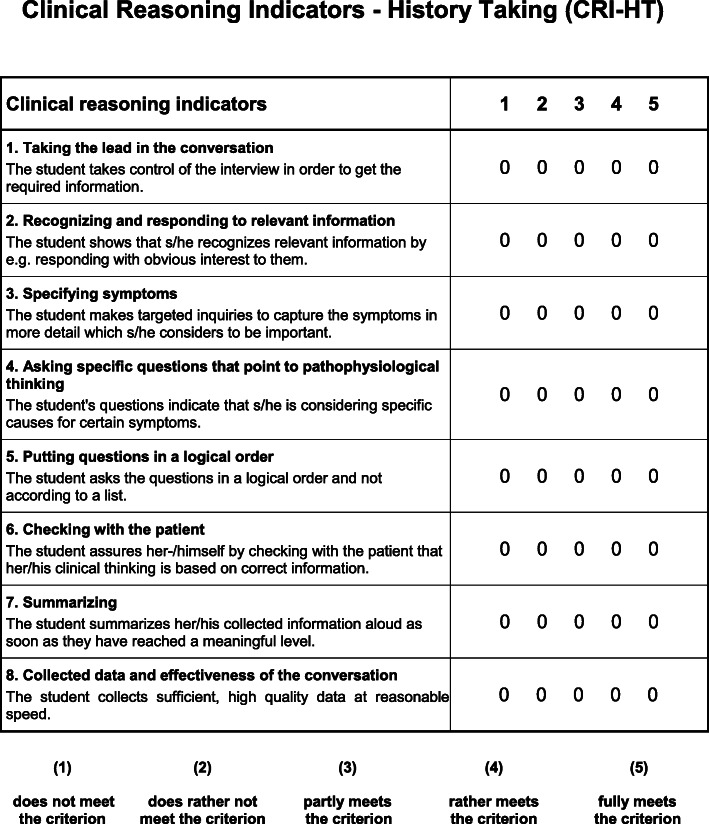
Clinical Reasoning Indicators - History Taking - Scale (CRI-HT-S)

In 2018, Haring et al. identified 13 relevant items which were abstracted from students’ observable behaviour during history taking by expert assessors in a qualitative study using a grounded theory approach [11]. We used eight of her original items, which were identified as clinical reasoning indicators and transformed them into a scale (Fig. 1), which we named Clinical Reasoning Indicators - History Taking Scale (CRI-HT-S). We included the following items, which describe student activities associated with language or language related behaviour and which can be quantitated: 1. Taking the lead in the conversation, 2. Recognizing and responding to relevant information, 3. Specifying symptoms; 4. Asking specific questions that point to pathophysiologic thinking, 5. Putting questions in a logical order, 6. Checking with patient, 7. Summarizing, and 8. Data gathering and efficiency. The remaining five items, which did not meet these criteria, e.g. body language [11], were not included in the scale. Each included item was operationalized with a short description and could be rated on a 5-point Likert scale: 1 = does not meet the criterion at all, 2 = rather does not meet the criterion, 3 = partly meets the criterion, 4 = rather meets the criterion, and 5 = fully meets the criterion. A maximum clinical reasoning ability sum score of 40 could be achieved for each individual conversation with one patient. Internal-rater reliability was scrutinized in a pilot by TH and SH assessing a sample of 10 videos. The agreement per item for this pilot was set to 80.0%. On this condition, an overall agreement for the evaluation of these 10 videos of .91 was reached between the two raters. Additionally, we calculated the intraclass correlation coefficient (ICC) for the whole questionnaire, which was .541 and can be described as “fair” [18]. The 325 videos were subsequently assessed by TH using this scale in a random and blinded order. The internal consistency (Cronbach’s alpha) of the CRI-HT-S in our study sample was .78. Because clinical reasoning is highly context specific, the Cronbach’s alpha of the CRI-HT-S for the different patient cases were also calculated: patient 1 = .70, patient 2 = .66, patient 3 = .63, patient 4 = .63, and patient 5 = .67.

In July 2017, 70 advanced medical students (semester 10 to 12, i.e. end of year five to end of year six of a six-year undergraduate medical training) from three medical schools of different size and location (University Hamburg, University Oldenburg, Technical University Munich) participated in the validated 360-degree assessment [17] which took place at the University Medical Center Hamburg-Eppendorf (UKE). The Technical University Munich provides a standard clinical curriculum following two years of pre-clinical studies. The universities Hamburg and Oldenburg teach medicine in vertically integrated courses with pre-clinical and clinical content being taught from year one. Neither of the universities teaches hypothesis-directed history taking or clinical reasoning. At the end of semester 10, students have finished all clinical rotations and afterwards, up to semester 12, students work full time on wards for a maximum of one year. Three students from Hamburg were excluded from the analysis because they had not reached their 10th semester. One student from Oldenburg and one student from Munich had to be excluded because of incomplete data sets. Eventually, data from 65 students were analyzed: *n =* 35 students from the University Medical Center Hamburg-Eppendorf, *n* = 5 students from the Carl von Ossietzky University of Oldenburg, and *n =* 25 students from the Technical University of Munich. Additionally, sociodemographic data of these 65 students (semester 10, *n* = 25; final year, *n* = 40) were collected. In total, 325 videos were obtained (*n = 125* from semester 10 and *n = 200* from final year). This study is part of a larger study [19]. The Ethics Committee of the Chamber of Physicians (Ethik-Kommission, Ärztekammer Hamburg), Hamburg, confirmed the innocuousness of the study and its accordance with the Declaration of Helsinki. Participation was voluntary and anonymized and participants consented to their participation (reference number: PV3649).

A factor analysis was conducted for the items of the questionnaire, which resulted in three factors. For statistical analysis, the means (*M*) of the different clinical reasoning indicators, of the three factors, and of the CRI-HT-S sum scores of the complete scale were calculated, respectively, per group of participants. Bonferroni correction for multiple testing was applied when necessary. Cohen’s d was calculated as measure for the effect size of significant differences. CRI-HT-S sum scores and means of the three factors were additionally calculated per patient and per group of participants. Statistical analysis included a two-way analysis of variance (ANOVA) to study differences between the groups of participants.

## Results

Of the 65 participants, 25 medical students were in semester 10 (age: 25.5 ± 2.2 years), 40 students were in their final year of undergraduate medical training (age: 26.4 ± 2.1 years), 37 participants were female and 28 were male. Table 1 shows the rotated factor matrix with the eight clinical reasoning indicators loading on three factors. Factor 1 includes three items comprising aspects of “Focusing questions”, factor 2 consists of three items, which depict “Creating context”, and factor 3 includes two items, which indicate “Securing information”. In Table 2, the overall means for the eight individual CRI-HT-S items and the three factors are shown, respectively. Means of all factors differ significantly (*p* < .001) with factor 1 reaching the highest (4.07 ± .47) and factor 3 reaching the lowest mean score (2.79 ± .83). “Taking the lead in the conversation” was the individual item with the highest score (4.36 ± .42) while “Summarizing” reached the lowest score (2.27 ± .93). The total mean score for the complete CRI-HT-S including all eight items was 28.96 ± 2.89.

**Table 1:** Rotated factor matrix with loadings

Indicators of clinical reasoning ability	Factor		
	1	2	3
Taking the lead in the conversation	.904	.046	.135
Putting questions in a logical order	.751	.375	.190
Specifying symptoms	.470	.364	.435
Asking specific questions that point to pathophysiological thinking	.204	.808	.056
Collected data and effectiveness	.071	.740	.447
Recognizing and responding to relevant information	.530	.597	-.101
Summarizing	.037	.024	.902
Checking with the patient	.217	.168	.810

**Table 2:** Clinical reasoning ability of all participating students according to factors

Indicators of clinical reasoning ability	*M ± SD*	*M ± SD*	Factor
Taking the lead in the conversation	4.36 ± .42	4.07 ± .47 *	**1**
Putting questions in a logical order	4.00 ± .47		
Specifying symptoms	3.86 ± .53		
Asking specific questions that point to pathophysiological thinking	3.87 ± .53	3.72 ± .43 *	**2**
Collected data and effectiveness of the conversation	3.62 ± .34		
Recognizing and responding to relevant information	3.67 ± .43		
Summarizing	2.27 ± .93	2.79 ± .83 *	**3**
Checking with the patient	3.30 ± .72		

*p < .001

With respect to the sum scores reached for the five different patients no significant differences could be detected between the patients (Table 3). When the mean scores for the three different factors were compared between the five patients, we found significantly lower mean scores (*p* ≤ .001) for factor 1 in patient 1 compared to the other four patients, and for patient 2 (man with fatigue and hemoptysis) versus patient 3 (woman with abdominal pain) and 5 (woman with rheumatoid arthritis and fever). Students from semester 10 showed significantly lower overall scores with medium effect size for factor 3 (*p* < .05, *d* = .69) and both items of this factor compared with students in their final year (Table 4). Even though students from semester 10 reached slightly lower CRI-HT-S sum scores for the individual patients their scores did not differ significantly from the CRI-HT-S scores reached by final year students (10th semester versus final year: patient 1: 26.16 ± 4.73 versus 27.22 ± 3.69; patient 2: 26.72 ± 4.21 versus 29.07 ± 3.00; patient 3: 29.84 ± 4.11 versus 30.68 ± 3.12; patient 4: 28.16 ± 3.94 versus 28.80 ± 3.15; patient 5: 29.56 ± 3.80 versus 31.20 ± 3.80).

**Table 3:** Total sum scores of indicators of clinical reasoning and factors of clinical reasoning of all participating students per simulated patient

Patient cases	Sum score	Factor 1	Factor 2	Factor 3
	*M ± SD*	*M ± SD*	*M ± SD*	*M ± SD*
Patient 1: 42-year-old female with palpitations	27.15 ± 4.16	3.55 ± .67***	3.89 ± .48*	2.42 ± .90*
Patient 2: 53-year-old male with fatigue and hemoptysis	28.17 ± 3.67	3.92 ± .58***	3.73 ± .54	2.62 ± .90*
Patient 3: 58-year-old female with abdominal pain	30.35 ± 3.52	4.45 ± .50***	3.68 ± .46	2.98 ± .99
Patient 4: 54-year-old male with flank pain	28.55 ± 3.46	4.09 ± .48**	3.59 ± .56	2.76 ± .85*
Patient 5: 36-year-old female with rheumatoid arthritis and fever	30.57 ± 3.85	4.36 ± .55	3.72 ± .47	3.17 ± 1.04

Factor 1: ***p ≤ .001: patient 1 versus patient 2, 3, 4, and 5; patient 2 versus patient 3 and 5, patient 3 versus patient 4. **p ≤ .01: patient 4 versus patient 5

Factor 2: *p < .05: patient 1 versus patient 3 and 4

Factor 3: *p < .05: patient 1 versus patient 3, 4 and 5; patient 2 versus patient 3 and 5; patient 4 versus patient 5

**Table 4:** Clinical reasoning ability of students from semester 10 and from the final year according to individual items and factors

Indicators of clinical reasoning ability	Semester 10 *M ± SD*	Final Year *M ± SD*	Semester 10 *M ± SD*	Final Year *M ± SD*	Cohen’s d	Factor
Taking the lead in the conversation	4.31 ± .45	4.39 ± .40	3.99 ± .42	4.12 ± .36	.33	**1**
Putting questions in a logical order	3.89 ± .48	4.07 ±. 46				
Specifying symptoms	3.78 ± .63	3.92 ± .46				
Asking specific questions that point to pathophysiological thinking	3.77 ± .61	3.94 ± .46	3.69 ± .39	3.74 ± .31	.14	**2**
Collected data and effectiveness of the conversation	3.57 ± .40	3.65 ± .30				
Recognizing and responding to relevant information	3.74 ± .42	3.64 ± .43				
Summarizing	1.98 ± .85*	2.46 ± .94	2.52 ± .68*	2.96 ± .74	.69	**3**
Checking with the patient	3.06 ± .71*	3.46 ± .70				

* *p* < .05: significantly different compared to final year

## Discussion

Assessing clinical reasoning as part of the clinical reasoning process is an important but difficult task. Faculty members have different frames of reference when translating observations into judgements, high levels of inference can occur while observing students, and the way by which judgements are transferred into a numerical rating system can vary [12]. The instrument (CRI-HT-S), which was developed from qualitatively identified clinical reasoning indicators [11] to assess clinical reasoning quantitatively during history taking, showed good internal consistency. This supports the use of the CRI-HT-S since clinical reasoning leads to the correct final diagnosis after history taking alone in 76.0% [15]. In general, our participating students reached the highest scores in the CRI-HT-S factor 1 (“Focusing questions”) including the items “Taking the lead in the conversation”, “Putting questions in a logical order”, and “Specifying symptoms”. We interpreted this as a sign that the students learned to apply specific patterns to organize patient information and relate it to their own knowledge in an expert way [20]. Rather than just applying a history taking scheme to their patient interviews the students were able to organize their history taking in a chief complaint driven way [21]. The item “Asking specific questions that point to pathophysiological thinking”, which received the third-highest rating and belongs to factor 2 (“Creating context”), demonstrates that the students, like experts, were able to intuitively generate a list of diagnostic hypotheses to a certain extent and to test them by analytic reasoning [22]. “Summarizing”, which is an important aspect of clinical reasoning during history taking to give the interviewer a chance to review the history for lack of clarity [23] was the only item with a score below satisfactory. Whether this relates to the way history taking is taught or is an effect of the time pressure students might have felt during the patient consultations remains to be investigated.

Students in their final year of undergraduate medial education showed significantly higher scores for factor 3 (“Securing information”) and for its two items “Summarizing” and “Checking with the patient”, respectively, than students from semester 10. This is an interesting finding which could reflect that medical students up to semester 10 are still taught history taking in the traditional way by obtaining the history in separate sequential categories (e.g. “history of the present illness”, “past medical history”, “review of systems” etc.) [24]. It has been shown over 40 years ago, that his method to teach history taking is deficient when used as the only teaching method [25]. The combination of content and process of history taking leads to better learning outcomes with respect to clinical reasoning [26, 27]. Students in their final year have more learning opportunities with real history taking while working full time on hospital wards. Therefore, their learning is less static and rather resembles problem-based learning tutorials [28]. This might be a reason why they achieved higher scores for factor 3 in our study.

With respect to the different patients, the highest scores were reached for factor 1 when an illness script based on the presenting complaint could be easily developed (e.g. patient 3, a 58-year-old woman with abdominal pain in the left lower quadrant) during history taking by pattern recognition [29]. The development of illness scripts can already be fostered by case-based learning in the early years of a curriculum [30], which is a standard didactic feature in undergraduate medical curricula [31]. Furthermore, the chief complaints of the patients in our study – palpitations, hemoptysis, abdominal pain, flank pain, and fever under immunosuppression, respectively, which are all learning objectives in the undergraduate curriculum – might have triggered clinical reasoning factors 1 (“Focusing questions”) and 2 (“Creating context”) with scores above 3.5 on a 5-point scale. This might be due to a focus on the pathophysiology of diseases, i.e. their biomedical details, which play a greater role for learners than for experts who have seen more cases with experience and rely to a greater extent on pattern recognition to develop an illness script [32]. The patients with the chief complaints “abdominal pain” and “fever under immunosuppression” received the highest overall CRI-HT-S scores. This might be a hint that these medical conditions could have played a more prominent role [33] in undergraduate medical education.

Our empirically developed CRI-HT-S was not validated in a separate study by, e.g., comparing it with validated instruments for the assessment of reasoning or with the clinical reasoning outcomes of the participants in our assessment, which clearly represents a major weakness. However, the CRI-HT-S seems to be a tool with consistent performance in discriminating learners’ clinical reasoning abilities across settings and shows an acceptable internal consistency of .78. The agreement between raters in a pilot test was .91 and ICC was “fair” (.541). However, it would have strengthened our study further, if a pilot test of the instrument had taken place with clinicians outside the team and if two to three raters had assessed all videos. Furthermore, different rater biases and aspects of rater variability have been described while assessing clinical skills, which e.g. include the raters’ background knowledge and skills that will substantially shape their interpretation of others’ behaviour [34, 35]. This strongly indicates that the CRI-HT-S should be used with a rater training including aspects of rater biases and anchors to transfer possible observations of the different clinical reasoning indicators into a numerical scale to receive results with good internal consistency. Another weakness of the CRI-HT-S is that the items “Specifying symptoms” and “Recognizing and responding to relevant information” showed factor loadings below 0.6. However, both factors did not show higher loadings for another factor and their content fitted well with factor 1 and factor 2, respectively. Since we were only sampling a small subset of patient cases (i.e. context), our ability to make broad interpretations of medical knowledge is limited. On the other hand, we took care of balanced case difficulty during case design. The strengths of our study are the high number of rated videos and the participation of students from more than one university supporting the generalizability of our results. Furthermore, this is – to our knowledge – the first study with an operationalized scale which enables supervisors to quantitatively measure clinical reasoning indicators.

## Conclusion

The empirically constructed CRI-HT-S could be applied with consistent performance and acceptable internal consistency to assess clinical reasoning indicators during history taking in videos of a simulated consulting hour with advanced medical students in the role of a beginning resident. Higher scores for the clinical reasoning factor “Creating context” could be observed in more advanced undergraduate medical students with longer clinical training. For patient cases, where an illness script could by easier developed, higher scores were achieved for the clinical reasoning factors “Focusing questions” and “Creating context”. Whether the CRI-HT-S has potential to be used as a scale in objective structured clinical examinations or in workplace-based assessments of clinical reasoning has to be investigated in further studies with larger student cohorts.

## Data Availability

All data and materials are available in the manuscript and can be obtained from the corresponding author upon request.

## Abbreviations

CRI-HT-S: Clinical Reasoning Indicators- History Taking-Scale
ICC: Intraclass correlation coefficient
TUM: Technical University of Munich
UKE: University Medical Center Hamburg Eppendorf
